# Application of a Screen-Printed Ion-Selective Electrode Based on Hydrophobic Ti_3_C_2_/AuNPs for K^+^ Determination Across Variable Temperatures

**DOI:** 10.3390/ijms252313204

**Published:** 2024-12-08

**Authors:** Zhixue Yu, Hui Wang, Yue He, Dongfei Chen, Ruipeng Chen, Xiangfang Tang, Mengting Zhou, Junhu Yao, Benhai Xiong

**Affiliations:** 1 State Key Laboratory of Animal Nutrition, Institute of Animal Sciences, Chinese Academy of Agricultural Sciences, Beijing 100193, China; 15831207256@163.com (Z.Y.); wanghui10@caas.cn (H.W.); heyueh@163.com (Y.H.); chenruipeng@caas.cn (R.C.); tangxiangfang@caas.cn (X.T.); zhoumengting@caas.cn (M.Z.); 2 College of Animal Science and Technology, Northwest A&F University, Xianyang 712100, China; 3 College of Animal Science and Technology, China Agricultural University, Beijing 100193, China; 4 Graduate School of Biomedical Engineering, The University of New South Wales, Sydney, NSW 2052, Australia; dongfei.chen@unsw.edu.au

**Keywords:** Ti_3_C_2_ Mxene, AuNPs, hydrophobic, K^+^-selective electrode, temperature sensor, hypokalemia

## Abstract

Monitoring potassium ion (K^+^) concentration is essential in veterinary medicine, particularly for preventing hypokalemia in dairy cows, which can severely impact their health and productivity. While traditional laboratory methods like atomic absorption spectrometry are accurate, they are also time-consuming and require complex sample preparation. Ion-selective electrodes (ISEs) provide an alternative that is faster and more suitable for field measurements, but their performance is often compromised under variable temperature conditions, leading to inaccuracies. To address this, we developed a novel screen-printed ion-selective electrode (SPE) with hydrophobic Ti_3_C_2_ Mxene and gold nanoparticles (AuNPs), integrated with a temperature sensor. This design improves stability and accuracy across fluctuating temperatures by preventing water layer formation and enhancing conductivity. The sensor was validated across temperatures from 5 °C to 45 °C, achieving a linear detection range of 10^−^⁵ to 10^−1^ M and a response time of approximately 15 s. It also demonstrated excellent repeatability, selectivity, and stability, making it a robust tool for K^+^ monitoring in complex environments. This advancement could lead to broader applications in other temperature-sensitive analytical fields.

## 1. Introduction

Potassium is one of the most abundant mineral elements in animal bodies, ranking third in content after calcium and phosphorus [1]. It plays a crucial role in cellular function, being involved in regulating osmotic pressure, signal transduction, acid-base regulation, nerve impulse transmission, and muscle contraction [2]. In cows, serum potassium levels are typically maintained within the range of 3.5 to 5.8 mM [3]. However, during the peripartum period, cows undergo significant physiological changes that can lead to unstable metabolic processes. Insufficient potassium intake during this time can result in postpartum hypokalemia, characterized by limb weakness, difficulty standing, noticeable muscle tremors, extended periods of lying down, and respiratory distress. Affected cows often experience reduced appetite and lactation, which can adversely affect their health and productivity. This condition can also increase the culling rate, leading to economic losses for cattle farms. Therefore, there is a critical need for a detection method that can rapidly and accurately measure potassium ions in cow serum.

Common methods for detecting serum potassium ions, such as atomic absorption spectrometry (AAS) and atomic fluorescence spectrometry (AFS), are known for their sensitivity and effectiveness. However, these techniques have several drawbacks, including the requirement for expensive instruments, skilled operators, complex sample preparation processes, and time consumption, resulting in high measurement costs. In contrast, electrochemical sensors have the advantages of high reproducibility, linear output, and low power consumption, providing good sensitivity and selectivity for routine analysis of different target analytes. In electrochemical sensors, ion-selective electrodes (ISEs) are promising for rapidly monitoring potassium ions due to their fast measurement speed and high sensitivity. The principle of ISEs involves an ion-selective membrane that selectively allows potassium ions to pass through while excluding other ions. The potential generated by the electrode follows Nernst’s response to the activity of potassium ions in the solution, allowing the concentration of potassium ions in the serum to be accurately determined by measuring the potential change. Solid contact ion-selective electrodes (SC-ISEs) are widely used in biological analysis due to their robust structure, portability, and suitability for miniaturization [4]. Various methods have been proposed to improve the performance of SC-ISEs, including the use of conductive polymers as intermediate layers between ion-selective membranes and conductive substrates, which serve as ion-electron transducers, improving potential stability and reducing detection limit [5,6]. However, a persistent challenge in SC-ISEs is the formation of water layers between the ion-selective membrane and the conductive substrate, leading to potential drift and a compromised detection limit [7]. Therefore, developing strategies to prevent the formation of water layers is crucial.

Mxenes, a new type of 2D transition metal material, offer a unique combination of properties, including a regular layered structure, variable elemental composition, excellent conductivity, and a large specific surface area [8]. These exceptional characteristics make Mxenes suitable for a wide range of applications, including lithium batteries [9], energy storage [10], electrocatalysis [11], and electrochemical sensing applications [12]. Among them, Ti_3_C_2_ Mxene stands out as an ideal electrode material due to its high conductivity, high specific surface area, excellent hydrophilicity, and ease of surface modification. Mxene’s two-dimensional lamellar structure and high electrical conductivity provide more channels for ion transport and charge transfer [13]. AuNPs are frequently employed in electrochemical sensors to increase their electroactive surface area and electron mobility [14]. Furthermore, the surface modification of Ti_3_C_2_ Mxene with AuNPs can significantly improve the stability, conductivity, and biocompatibility, making it more effective in electrochemical sensors [15,16].

Despite the promising properties, Ti_3_C_2_ Mxene’s inherent hydrophilicity, due to its polar end group, presents a challenge in preventing water layer formation on the electrode surface. To address this, Ti_3_C_2_ Mxene can be treated with octadecyltrichlorosilane (OTS), a reagent that imparts hydrophobicity by introducing long-chain alkyl groups, thus preventing water layer formation and improving electrode performance [17,18].

SPEs are disposable sensors produced via screen printing technology, where various inks are printed on ceramic or plastic surfaces. SPEs offer several advantages, including the incorporation of a (two) three-electrode system in a miniaturized flat structure, enabling their use either once or repeatedly, thereby minimizing contamination and memory effects. Additionally, SPEs can analyze samples volume as small as tens of microliters. They are mass-producible, and the working electrode surface can be easily modified, providing many possibilities for electrode optimization. These modifications include immobilized biological components, organic–metal catalysts, carbon nanotubes, nano-metal particles, etc. [19,20]. Therefore, SPE is selected to determine potassium ion concentration.

The performance of ISE is influenced by temperature [21]. Temperature sensors, capable of real-time monitoring of environmental temperature changes, hold significant value in electrochemical detection. By integrating temperature sensors into electrochemical detection systems, real-time temperature data can be obtained, and the performance of electrochemical sensors can be calibrated and compensated based on temperature changes. This not only helps to improve the accuracy and stability of electrochemical detection, but also broadens the application scope of these sensors, making them more adaptable to complex and dynamic environmental conditions.

In summary, with the continuous development of electrochemical detection technology and the continuous expansion of application fields, the demand for improved accuracy and stability has increased. Under variable temperature conditions, traditional electrochemical sensors often face many challenges in measuring K^+^ concentration, such as performance changes caused by temperature fluctuations and interference from non-target ions. The screen-printed ion-selective electrode based on hydrophobic Ti_3_C_2_/AuNPs proposed in this study effectively prevents interference from non-target ions and enhances the sensitivity and stability of the electrode by introducing hydrophobic Ti_3_C_2_ Mxene and AuNPs. The experimental results indicate that the sensor can achieve a timely response to K^+^ concentrations of 10^−6^–10^−1^ M within a temperature range of 5–45 °C. The neural network linear regression method was used to process and model the data, and the correlation coefficient between the predicted values and the true values was 0.9921, indicating a good correlation.

## 2. Results and Discussion

### 2.1. Characterization of Electrode Preparation Process

**Figure 1:**
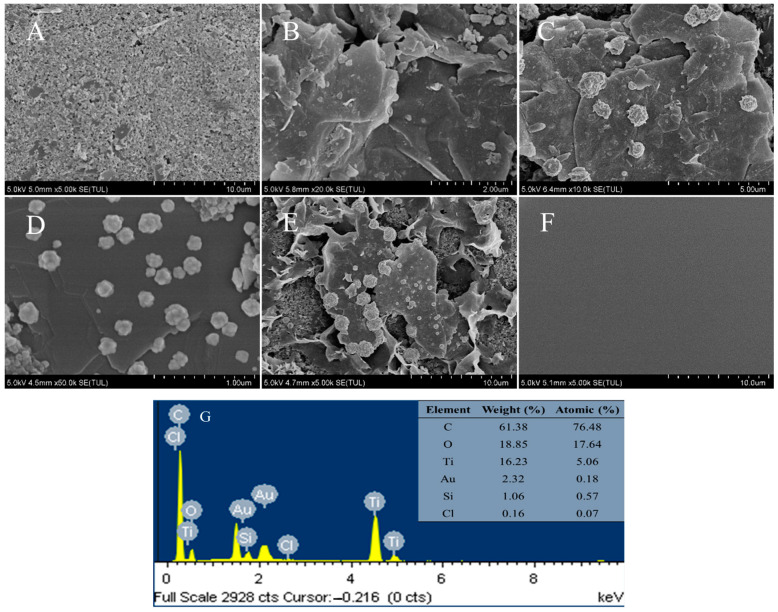
SEM images of bare SPE (**A**), SPE/Ti_3_C_2_ (**B**), SPE/Ti_3_C_2_/AuNPs (**C**,**D**), SPE/OTS-Ti_3_C_2_/AuNPs (**E**), SPE/OTS-Ti_3_C_2_/AuNPs/K-ISM (**F**), and EDS mapping analysis (**G**).

Figure 1 characterizes the scanning electron microscopy (SEM) images of the SPE’s surface morphology at different modification stages. As shown in Figure 1A, coarse amorphous carbon particles can be observed on the surface of the SPE, with paste and flake shapes. When Ti_3_C_2_ Mxene, AuNPs, OTS, and K-ISM were deposited on the SPE, the electrode surface morphology was different. As shown in Figure 1B, Ti_3_C_2_ Mxene can be observed on the electrode surface as ultra-thin multilayer nanosheets. The stacking of thin nanosheets effectively increases the specific surface area of the electrode, providing more active sites for the deposition of AuNPs [22]. Figure 1C,D show the electrode surface after the deposition of AuNPs. It can be clearly seen that the Ti_3_C_2_ Mxene surface has granular protrusions, indicating that AuNPs have been successfully deposited on the electrode surface [23]. Figure 1E shows the electrode surface treated with OTS hydrophobic treatment, which is covered with some crumpled material. Figure 1F shows the SEM image after K-ISM modification. Due to the non-conductivity of K-ISM, the electrode surface appears as a smooth surface. Thus, each material has been successfully modified on the surface of SPE.

Figure 1G shows the energy-dispersive spectroscopy (EDS) analysis results of Ti_3_C_2_ Mxene, AuNPs, and OTS modified on the surface of SPE. The modified electrode surface consisted of 61.38 wt% C, 18.85 wt% O, 16.23 wt% Ti, 2.32 wt% Au, 1.06 wt% Si, and 0.16 wt% Cl. The presence of Si and Cl elements confirmed the successful fixation of OTS on the electrode surface. In these cases, SEM images and EDS mapping analysis showed a uniform distribution of elements on the sample surface, indicating successful electrode surface modification.

### 2.2. Electrochemical Characterization of the Fabricated Sensor

**Figure 2:**
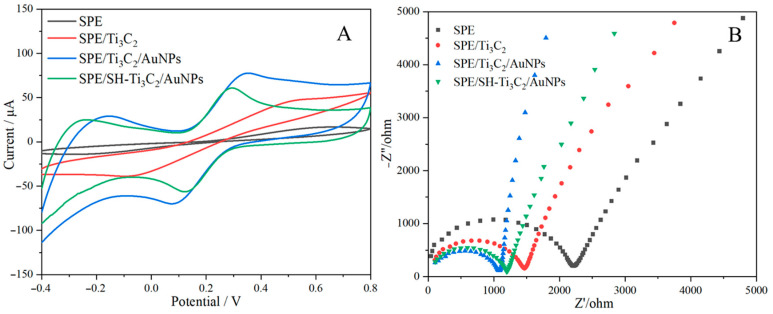
CV (**A**) and EIS (**B**) of different modification processes at 5.0 mM [Fe(CN)_6_]^3−/4−^ solutions.

The electrochemical behavior of SPE modified with Ti_3_C_2_ Mxene, AuNPs, and OTS was analyzed using cyclic voltammetry (CV) and electrochemical impedance spectroscopy (EIS) in a 5.0 mM [Fe(CN)_6_]^3−/4−^ solution. As illustrated in Figure 2A, the unmodified SPE exhibited low peak redox values due to its limited surface area and poor electrical conductivity. Upon modification with Ti_3_C_2_ Mxene, the peak current increased, attributed to the high conductivity and the large specific surface area of MXene, which enhanced both electrode current and capacitance [24]. Further modifications with AuNPs significantly improved the electrode’s sensitivity, owing to the synergistic interaction between AuNPs and Ti_3_C_2_ Mxene, which accelerated electron transfer and increased the effective surface area [23]. However, after applying OTS, the current response slightly decreased, due to the partial obstruction of electron transfer caused by the deposition of a single OTS layer on the electrode surface [25]. Despite the minor reduction, the current change was minimal, suggesting that hydrophobically-treated Ti_3_C_2_/AuNPs can effectively serve as an intermediate layer.

In the EIS analysis, the high-frequency semicircle region of the Nyquist plot represents the electron transfer process, with the diameter of the semicircle corresponding to the charge transfer resistance (*R_ct_*), which indicates the kinetic properties of the redox probe ([Fe(CN)_6_]^3−/4−^) during electron transfer [26]. As shown in Figure 2B, the bare SPE exhibited the largest semicircle, with an *R_ct_* value of 2209 Ω. By loading Ti_3_C_2_ Mxene, the *R_ct_* value was reduced to 1465 Ω, possibly due to the excellent electrical conductivity of Ti_3_C_2_ Mxene, which accelerates the electron transfer rate [27]. A significantly smaller semicircle (1084 Ω) was seen when AuNPs were added to the electrode surface, and this was explained by the Ti_3_C_2_/AuNPs nanocomposite’s superior electrical activity and conductivity [13]. When OTS continued to incubate on the electrode for 6–8 min, because OTS had a certain inhibitory effect on electron transfer, the *R_ct_* value (1203 Ω) increased slightly [25], which had little impact on the measurement results. This was consistent with the above CV results. The above data confirmed the successful fabrication of the sensor.

### 2.3. Raman Spectroscopy

**Figure 3:**
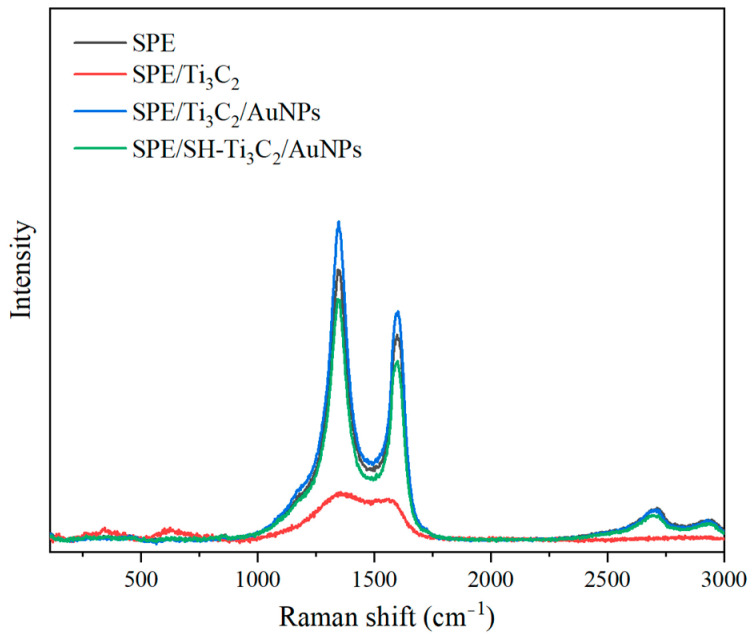
Raman spectra of different modification processes.

The characteristic Raman spectra of the electrode surface are shown in Figure 3. The Raman peaks on the bare SPE surface are mainly distributed in the D (1350 cm^−1^) and G (1579 cm^−1^) bands, which are typical graphite defects [28,29]. Raman spectroscopy of Ti_3_C_2_ MXene shows a strong Ti-C peak at 120–600 cm^−1^, with no obvious peak above 600 cm^−1^, consistent with previous studies [30]. After modification with AuNPs, the intensity ratio of D-band to G-band (I_D_/I_G_) decreased from 0.76 to 0.71, which was due to the increased number of nanoparticle layers on the electrode surface, resulting in the reduced I_D_/I_G_ intensity [31]. This indicates the successful deposition of AuNPs onto the Ti_3_C_2_ MXene layer. When the Ti_3_C_2_/AuNPs surface was modified with OTS, weak bands appeared due to methyl groups at the end of the alkyl chain [32]. 

### 2.4. Test of Contact Angle

**Figure 4:**
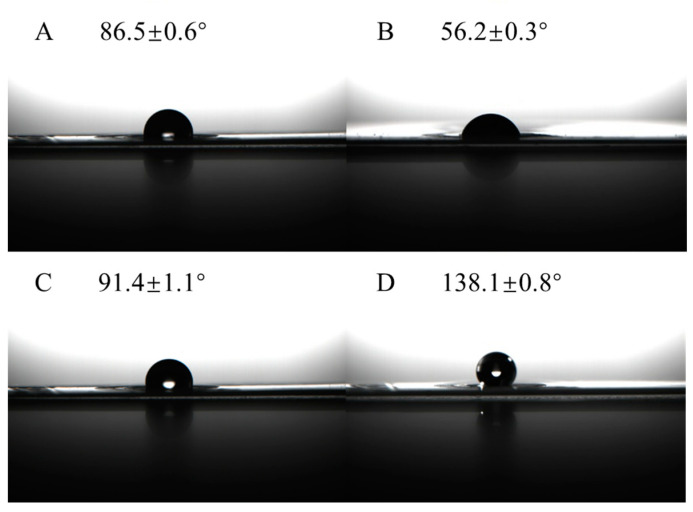
Contact angle measurements of bare SPE (**A**), SPE/Ti_3_C_2_ (**B**), SPE/Ti_3_C_2_/AuNPs (**C**), and SPE/OTS- Ti_3_C_2_/AuNPs (**D**).

Figure 4 presents the contact angles measurements for the bare SPE, SPE/Ti_3_C_2_, SPE/Ti_3_C_2_/AuNPs, and SPE/OTS-Ti_3_C_2_/AuNPs. As shown in Figure 4A, the bare SPE exhibited a contact angle of 86.5°, indicating a nearly hydrophobic surface. When the SPE was modified with Ti_3_C_2_ Mxene (Figure 4B), the contact angle was reduced to 56.2°, due to the hydrophilic nature of Ti_3_C_2_ Mxene. After the addition of AuNPs (Figure 4C), the contact angle increased slightly to 91.4°, suggesting a mild hydrophobic effect, similar to the lotus leaf phenomenon caused by AuNPs [33]. Finally, the surface of SPE/Ti_3_C_2_/AuNPs became more hydrophobic after the modification with OTS, which forms a self-assembling monolayer. This resulted in the largest contact angle, approximately 138.1°, indicating the formation of a hydrophobic conversion layer.

### 2.5. Water Layer Test

**Figure 5:**
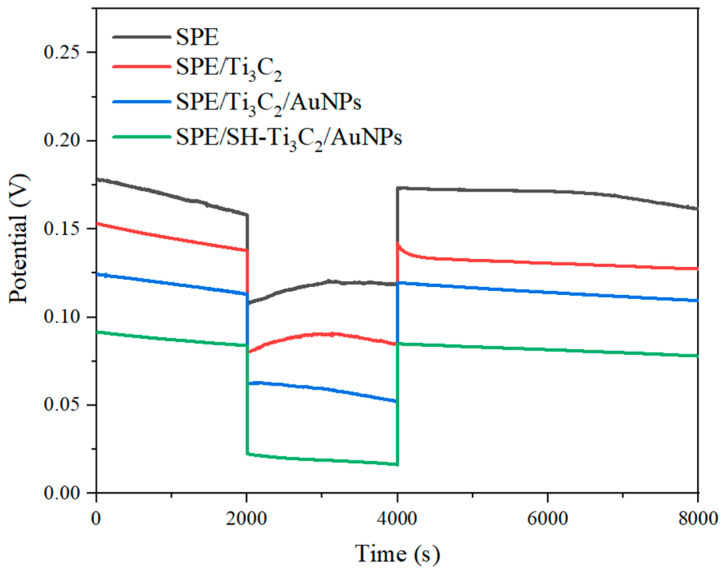
Different modification processes were tested in water layer in 10^−2^ M KCl and 10^−2^ M CaCl_2_.

The formation of a water layer between the selective film and the conductive substrate in solid-state ISE can significantly affect potential stability, posing a major limitation for solid-state sensors [34]. A vital component of studying solid-state ISE performance is the water layer testing experiment. Potential drift could happen when the electrode is moved from the main ion solution into the interfering ion solution if a layer of water forms [35]. In this study, the effects of water layers on the potential drift of SPE/K-ISM, SPE/Ti_3_C_2_/K-ISM, SPE/Ti_3_C_2_/AuNPs/K-ISM, and SPE/OTS-Ti_3_C_2_/AuNPs/K-ISM were examined, with results shown in Figure 5. After measuring the potential in a 10^−2^ M KCl solution, the electrode was taken out and put in a 10^−2^ M CaCl_2_ solution before being returned to the 10^−2^ M KCl solution, and the potential change was recorded. Compared with the other three sensors, the potential drift of SPE/OTS-Ti_3_C_2_/AuNPs/K-ISM was the smallest, because the hydrophobic-treated Ti_3_C_2_/AuNPs layer had hydrophobic properties, thus avoiding the formation of water layers. In contrast, SPE/K-ISM formed a water layer without a solid conductive layer, resulting in the greatest potential drift. These results indicated that using hydrophobic-treated Ti_3_C_2_/AuNPs as solid conductive layers effectively reduces the occurrence of a water layer between the transition layer and ISM.

### 2.6. Chronopotentiometry

**Figure 6:**
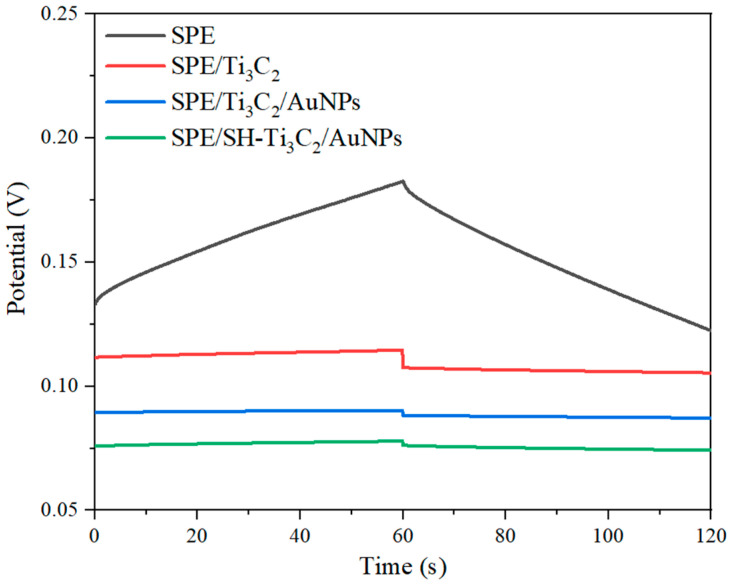
Chronopotentigrams of different modified electrodes at 10^−2^ M KCl.

The short-term potential stability of K-ISE modified with hydrophobic Ti_3_C_2_/AuNPs as an intermediate layer was evaluated using chronopotentiometry at a constant current of ±1 nA for 60 s in a 10^−2^ M KCl solution [36]. The resulting chronopotentiograms are shown in Figure 6, reflecting the K^+^ membrane’s ability to adapt to changes in ion concentration. During both cathodic and anodic polarization, the potential drift of K-ISE modified with Ti_3_C_2_ and AuNPs was much lower than that of unmodified K-ISE, which was similar to previously reported results [23]. The potential change rate ΔE/Δt = I/C (where ΔE is the potential change, Δt is the time change, I is the applied current, and C is the low-frequency capacitance) was used to evaluate the stability of the electrode [37]. The potential change rates of SPE/Ti_3_C_2_/K-ISM, SPE/Ti_3_C_2_/AuNPs/K-ISM, and SPE/OTS-Ti_3_C_2_/AuNPs/K-ISM were 48, 14, and 31 μV/s, respectively. They were all smaller than SPE/K-ISM (993 μV/s), indicating that the modified electrode had potential stability. This effect can be explained by the high electrical conductivity of the transfer layer. The hydrophobic treatment and incorporation of Ti_3_C_2_/AuNPs greatly improved the potential stability of the all-solid-state K-ISE.

### 2.7. Potential Characteristic

**Figure 7:**
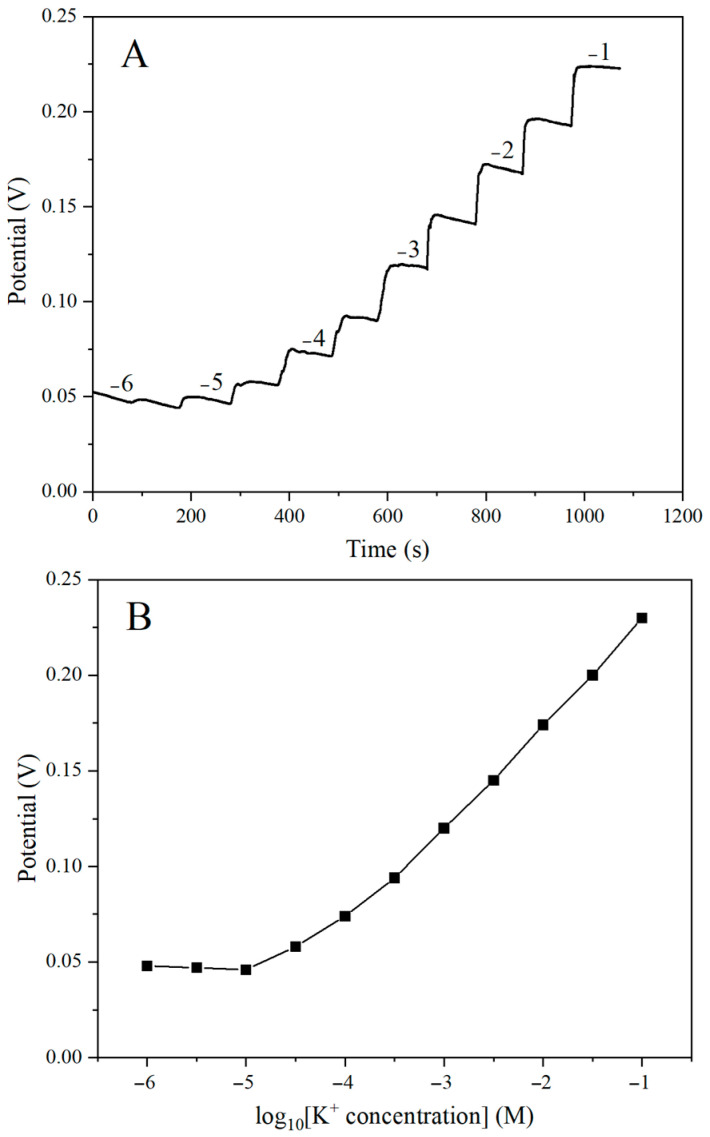
(**A**) Potential response of SPE/OTS-Ti_3_C_2_/AuNPs/K-ISM with increasing concentration of K^+^ and (**B**) calibration curve.

To evaluate the sensitivity of the electrode, K-ISE was tested in a solution containing different concentrations of K^+^ (10^−6^–10^−1^ M) for potential response measurements. Figure 7 is the potential response diagram and calibration curve, respectively. It can be seen that the potential increased with the increase in K^+^ concentration. The signal of the electrode system was stable, the noise was small, and the response time was fast, about 10–20 s, which was similar to the research of Miller et al. [38]. The electrode exhibited a linear response in the concentration range of 10^−5^ to 10^−1^ M, with a slope of 50.0 ± 1.6 mV/decade and a detection limit of 10^−5.2^ M with the correlation coefficient (R^2^) of 0.9863.

### 2.8. Detection Performance of Temperature Sensors

#### 2.8.1. Resistance Rate Response

**Figure 8:**
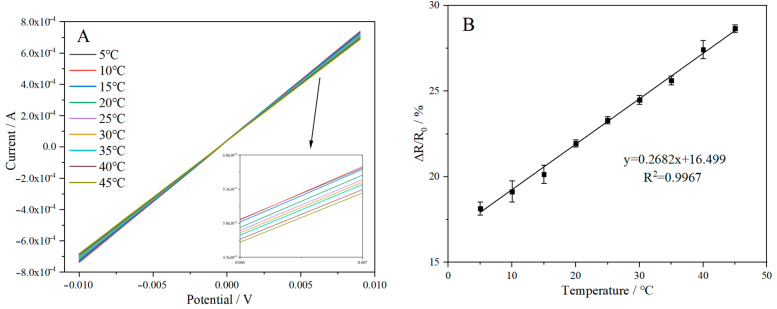
(**A**) LSV and (**B**) resistance change rate of temperature sensor at 5–45 °C.

Figure 8A shows the linear scanning voltammetry (LSV) test of the sensor at 5–45 °C with a 5 °C interval. The sensor exhibited good stability across this temperature range, with resistance gradually increasing as the temperature rose. The main reason is that as the temperature increases the atomic or molecular vibrations inside the temperature-sensitive material, which can interfere with the movement of charge carriers, leading to an increase in resistance. To quantify this behavior, the rate of change in resistance can be found using the formula ∆R/R_0_ = (R – R_0_)/R_0_, where R represents the real-time resistance at a given temperature and R_0_ is the reference resistance at 25 °C. The resistance change rate curve with temperature is shown in Figure 8B, which reveals a linear increase in resistance change with temperature. The high fitting degree (R^2^ = 0.9967) indicates that the sensor can accurately detect temperature changes, confirming its potential as a high-resolution temperature sensor.

#### 2.8.2. Effect of Temperature and K^+^ Concentration on Electrical Signal

**Figure 9:**
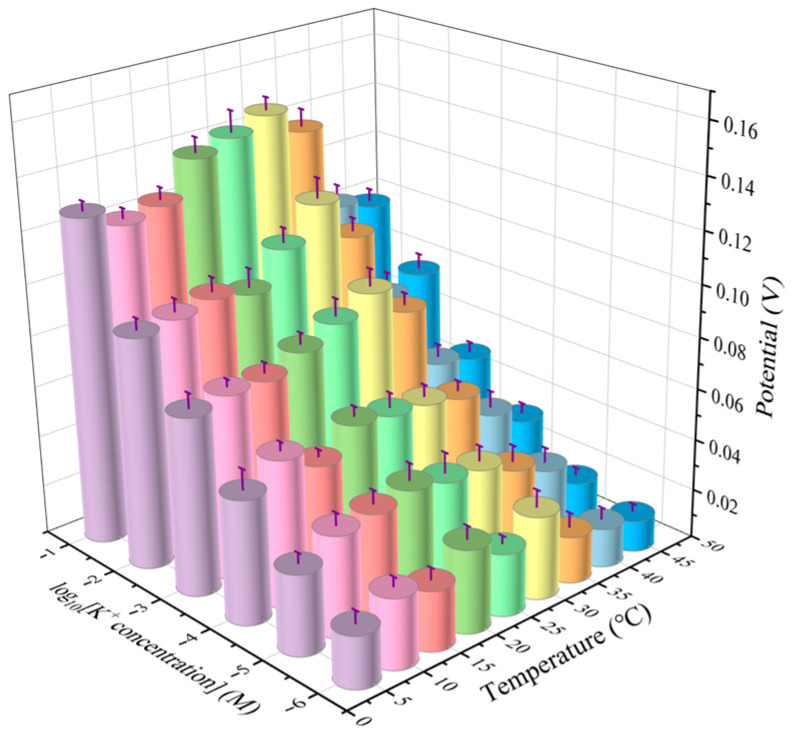
Potential of SPE/OTS-Ti_3_C_2_/AuNPs/K-ISM at different temperatures and K^+^ concentrations.

Based on the above research, the electrochemical sensor SPE/OTS-Ti_3_C_2_/AuNPs/K-ISM demonstrated a good linear range for K^+^ concentration detection. However, in practical applications, temperature fluctuations in the detection environment can reduce accuracy. To address this, a temperature sensor was developed and integrated onto the surface of the SPE, forming an electrochemical sensor array combined with potassium ion-selective sensors. This integration improves the accuracy and reliability of detection. A neural network linear regression method was employed for data processing and modeling to correct for the influence of temperature on the electrical signal. Figure 9 shows the electrical signal changes in the sensor at different temperatures and K^+^ concentrations. It can be seen that the potential response increased with the increase in K^+^ concentration at different temperatures, and the detection performance was best at 25–30 °C. When the temperature exceeded 35 °C, the electrochemical response of the sensor relatively reduced, which may be due to the influence of temperature on the ion activity coefficient, changing the relationship between ion activity and concentration [39]. In addition, the increase in temperature can also affect the stability and activity of ion carriers on the ISM, and exacerbate the impact of interfering substances in the solution on the electrode. Some interfering substances may compete with K^+^ to bind to the ISM, leading to a decrease in electrode selectivity.

#### 2.8.3. Data Preprocessing

To ensure accurate and reliable data selection for modeling, data preprocessing is required. Additionally, to eliminate the effects of different orders of magnitude and improve the convergence performance of the neural network, it is necessary to normalize the data according to the following formula:

$$Y={log}_{10}(C)/6$$

$$X=[1,t,t^{2},p,p^{2},t*p]$$

t = T/50, p = P∗5

where C is the concentration of K^+^, T and P are the actual temperature and electrical signal, and t and p are the data after normalization.

#### 2.8.4. Artificial Neural Network Model

**Figure 10:**
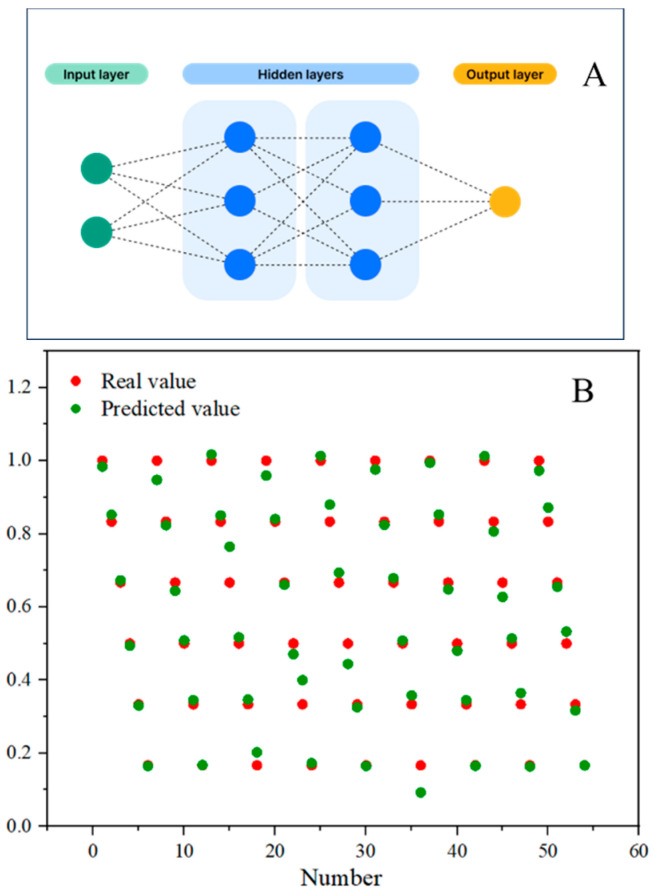
(**A**) The structure of the artificial neural network and (**B**) the calculated real and predicted values.

The artificial neural network model is a nonlinear modeling tool, which can be defined as a black box, consisting of a series of equations that produce outputs from given inputs after calculations. The structure of the neuron model is shown in Figure 10A, which is divided into three parts: input layer, hidden layer, and output layer. In this model, the X matrix is the input layer, with temperature (5 °C, 10 °C, 15 °C, 20 °C, 25 °C, 30 °C, 35 °C, 40 °C, 45 °C) as columns, and K^+^ concentration (10^−6^ M, 10^−5^ M, 10^−4^ M, 10^−3^ M, 10^−2^ M, 10^−1^ M) as rows, forming a matrix with nine columns and six rows. The Y matrix is the output layer, consisting of 1 column and 54 rows. The X matrix data are randomly divided into three groups: 70% for training samples, 15% for testing samples, and 15% for verification samples. Figure 10B compares the predicted values with the true values, yielding a correlation coefficient of R = 0.9921, indicating a good correlation between the true and predicted values. These results demonstrate that the artificial neural network model can be used to accurately predict the effects of different temperatures and K^+^ concentrations on the detected signal.

### 2.9. Stability and Selectivity

**Figure 11:**
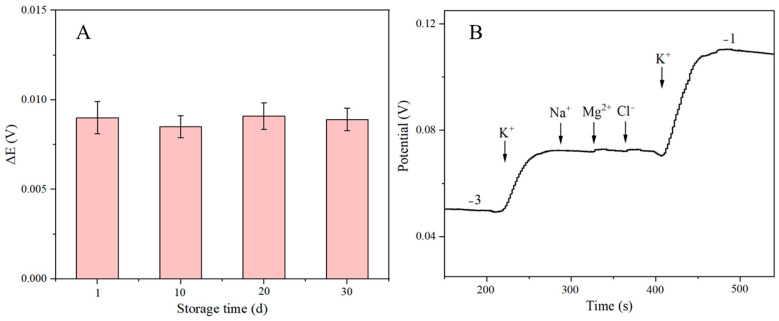
(**A**) Stability and (**B**) selectivity of the SPE/OTS-Ti_3_C_2_/AuNPs/K-ISM.

The stability of the SPE/OTS-Ti_3_C_2_/AuNPs/K-ISM sensor was assessed by storing the electrode under the same conditions (at 4 °C in a dry environment) for 30 days. Measurements were taken every ten days to calculate the potential difference, and the results are shown in Figure 11A. The data indicate that the potential of the sensor changed minimally over 30 days, demonstrating satisfactory long-term stability.

The selectivity or anti-interference of the sensor, is important for reliable performance. To evaluate selectivity, tests were conducted in the presence of potential interferents such as Na^+^, Mg^2+^, and Cl^−^. The selective test was performed using chronopotentiometry at an applied voltage of 0.3 V. As shown in Figure 11B, the injection of 10^−2^ M K^+^ solution resulted in a rapid, high potential response, while the addition of 10^−1^ M concentrations of interfering substances (Na^+^, Mg^2+^, and Cl^−^) had little effect on the potential response. This indicates that the addition of high concentrations of interfering substances did not affect the sensing performance in detecting K^+^. The results demonstrate that the sensor is highly selective for K^+^, with negligible interference from other ions. Additionally, as shown in Figure S1, the sensor’s response was unaffected by light and gas interference, further confirming its robustness. 

### 2.10. Comparison of Different K-ISEs

Our design provides a reliable method for K^+^ detection, as demonstrated in the comparison with other studies (Table 1). The developed sensor (SPE/OTS-Ti_3_C_2_/AuNPs/K-ISM) exhibits a larger detection range and lower detection limits than other works, extending well beyond the typical potassium ion concentrations found in the cow’s blood. This sensor eliminates the need for complex sample pretreatment before detection, enabling rapid and accurate determination of blood K^+^ concentration in dairy cows.

### 2.11. Determination of K^+^ in Samples

The accuracy of the sensor (SPE/OTS-Ti_3_C_2_/AuNPs/K-ISM) was studied by measuring K^+^ concentration in cow blood samples. The samples were pretreated as described in Section 2.5, with known amounts of K^+^ added to the blood samples. The measurement results are presented in Table 2. The recoveries of K^+^ samples ranged from 96.5% to 99.2%, with relative standard deviations (RSD, n = 3) below 6.50%. This method proved the accuracy and reliability of the sensor and provided a practical method for the determining of K^+^ concentrations in the serum of dairy cows.

## 3. Experimental

### 3.1. Reagents and Materials

Ti_3_C_2_ Mxene was ordered from Xianfeng Nano Technology Co., Ltd. (Nanjing, China). Valinomycin and tetrahydrofuran (THF) were purchased from Shanghai Jizhi Biochemical Technology Co., Ltd. (Shanghai, China). Shanghai Yuanye Biotechnology Co., Ltd. (Shanghai, China) supplied the polyvinyl chloride (PVC), 2-Nitrophenyl octyl ether (O-NPOE), and sodium tetrakis [3,5-bis (trifluoromethyl) phenyl] borate (NaTFPB). OTS, ethanol, and HAuCl_4_ were provided by Macklin Inc. (Shanghai, China). We purchased KCl, NaCl, CaCl_2_, and MgCl_2_ from Sigma Aldrich (Beijing, China). Ningbo Mxense Bio-Tech Co., Ltd. (Ningbo, China) provided the SPE, which had a 2.5 mm working electrode diameter. Polydimethylsiloxane (PDMS) was purchased from Hangzhou Weisichuang Tech Co., Ltd. (Hangzhou, China). PI film, temperature-sensitive ink (SEND-T02A), and conductive silver paste (BASE-CD01) were provided by Shanghai Mifang Electronic Tech Co., Ltd. (Shanghai, China).

### 3.2. Instruments and Measurements

Electrochemical experiments, such as cyclic voltammetry (CV) and electrochemical impedance spectroscopy (EIS), were conducted using a traditional three-electrode system on the CHI 760E electrochemical workstation (Shanghai Chenhua Instrument Co., Ltd., Shanghai, China). The working electrode was the SPE (SPE/OTS-Ti_3_C_2_/AuNPs/K-ISM). Ag/AgCl electrode, and platinum electrodes were used as the reference and counter electrodes, respectively. Field emission scanning electron microscopy (FE-SEM) images were obtained using a field emission scanning electron microscopy analyzer (FE-SEM SU8040, Tokyo, Japan). Raman spectrum was collected using Renishaw via an imaging microscope (532 nm diode and Ar ion laser). The contact angle measurement device (CSCDIC-100, Dongguan Shengding Precision Instrument Co., Ltd., Dongguan, China) was used to determine the surface hydrophobicity of the modified SPE. The temperature sensor was printed by a microelectronic printer (Shanghai Zhongbin Technology Co., Ltd., Shanghai China). The temperature was controlled by a heated magnetic stirrer (SCI280-Pro, Tuohe Electromechanical Technology (Shanghai, China) Co., Ltd., Shanghai, China).

### 3.3. Preparation of Electrode

**Figure 12:**
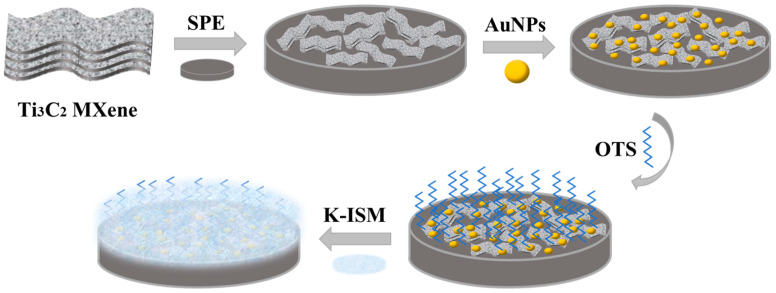
The preparation of SPE/OTS-Ti_3_C_2_/AuNPs/K-ISM.

Figure 12 illustrates the SPE/OTS-Ti_3_C_2_/AuNPs/K-ISM preparation procedure. Before modification, a cyclic potential scan was performed on the bare SPE within a potential window between −0.2 V and +1.2 V in a 2.5 mM [Fe(CN)_6_]^3−/4−^ solution to remove impurities from the electrode surface. The scanned SPE was rinsed with ultrapure water and then dried with nitrogen gas for later use. Subsequently, the working area of the electrode surface was covered with 20 μL Ti_3_C_2_ MXene solution (1.0 mg/mL) and dried under an infrared lamp to remove the solvent and obtain the SPE/Ti_3_C_2_. AuNPs were electrodeposited onto the SPE/Ti_3_C_2_ surface using cyclic voltammetry, by immersing SPE/Ti_3_C_2_ in 5 mL of 0.3 mM HAuCl_4_ solution and scanning for 8 cycles at a rate of 100 mV/s within the potential range of −0.2 to +1.2 V to form the SPE/Ti_3_C_2_/AuNPs. The electrode surface was then cleaned with ultra-pure water and dried at room temperature. OTS and ethanol were mixed at a ratio of 1:99, and then the electrode was soaked in it for 6–8 min for hydrophobic treatment. After removal, the surface was washed with ethanol, and SPE/OTS-Ti_3_C_2_/AuNPs were obtained after complete drying.

The mixture of K-Ion-Selective Membrane (K-ISM) consisted of 1.4% K^+^ carrier (valinomycin), 0.5% NaTFPB, 65.4% O-NPOE, and 32.7% PVC to form a 200 mg mixed membrane solution, and after that, 2 mL of THF was used to dissolve the mixed solution of the membrane material solution. In total, 20 μL K-ISM was added to the bare electrode SPE, SPE/Ti_3_C_2_, SPE/Ti_3_C_2_/AuNPs, and SPE/OTS-Ti_3_C_2_/AuNPs, respectively. This was added in increments of 5 μL each time, followed by the addition of one drop of THF after complete volatilization. The prepared electrodes were represented as SPE/K-ISM, SPE/Ti_3_C_2_/K-ISM, SPE/Ti_3_C_2_/AuNPs/K-ISM, and SPE/OTS-Ti_3_C_2_/AuNPs/K-ISM, respectively. After covering the electrodes with K-ISM, the solvent was evaporated at room temperature for 24 h. Before each measurement, the electrodes were adjusted in 0.01 M KCl solution for 3 h.

### 3.4. Preparation of Temperature Sensor

**Figure 13:**
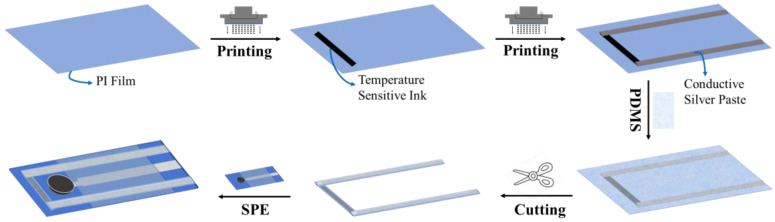
The preparation process of the temperature sensor.

To study the effect of temperature on the sensor, a fully printed temperature sensor was fabricated using inkjet printing technology, consisting of a temperature-sensitive layer and an electrode layer. Since the resistance of temperature-sensitive materials varies with temperature, the resistance change rate was measured by controlling temperature changes. The temperature sensor was fabricated on a PI film, and the preparation process is shown in Figure 13. First, the temperature-sensitive ink was printed in strips of 10 mm × 1 mm via inkjet printing on PI film. After heating at 150 °C for 10 min, a temperature-sensitive layer was formed. Then, the conductive silver paste was coated on both ends of the temperature-sensitive layer as a wire via dispensing and placed in an oven at 120 °C for 30 min. After removal, the surface was hydrophobic-treated with PDMS to ensure isolation between the sensor and the detection liquid. Then, the prepared temperature sensor was cut and pasted onto the surface of SPE, so that it could be detected simultaneously with SPE/OTS-Ti_3_C_2_/AuNPs/K-ISM.

### 3.5. Sample Measurement

Blood samples were obtained from the tail veins of cows at the China–Israel Demonstration Dairy Farm (Beijing, China). Following a 5 min centrifugation at 5000 rpm at room temperature, the samples were divided, and the supernatant was adjusted with 0.1 M Tris-HCl buffer (pH 7.4). The Chinese Academy of Agricultural Sciences (Beijing, China) Animal Care and Use Committee approved the animal process (IAS2021-237).

## 4. Conclusions

In this study, a novel electrochemical sensor, SPE/OTS-Ti_3_C_2_/AuNPs/K-ISM, was successfully developed by integrating a temperature sensor on the surface of SPE. The sensor was evaluated for its ability to detect K^+^ concentrations under varying temperature conditions. Firstly, the incorporation of hydrophobic Ti_3_C_2_ Mxene significantly enhanced the electrode’s anti-interference ability, allowing it to maintain a stable electrochemical response in complex environments. Secondly, the high catalytic activity and stability of AuNPs enabled the electrode to have higher sensitivity and lower detection limits (10^−5.2^ M) for K^+^ concentration measurement. Across the temperature range of 5–45 °C, the electrodes demonstrated high sensitivity and accuracy, with a broad linear range of 10^−5^–10^−1^ M and a rapid response time of approximately 15 s. In addition, the electrode exhibited good repeatability and selectivity. In summary, the new electrochemical sensor, which integrates a temperature sensor with SPE/OTS-Ti_3_C_2_/AuNPs/K-ISM, offers significant advantages and potential for measuring K^+^ concentration in complex environments and under different temperature conditions.

## Figures and Tables

**Table 1:** Performance comparison of SPE/OTS-Ti_3_C_2_/AuNPs/K-ISM with other sensors.

Sensor	Linear Range (M)	Response Time (s)	Detection Limit (M)	Ref.
PG K/ISE	10^−5^~10^−2^	20	10^−5.65^	[38]
Valinomycin-doped K-ISE	10^−3.1^~10^−1^	-	10^−3.1^	[40]
MoS_2_-based K-SC-ISE	10^−5^~10^−2^	-	10^−5.5^	[41]
K-SC-ISE	10^−4.5^~10^−1^	10	10^−4.5^	[42]
SPE/PANI/V	10^−5^~1	-	10^−5.8^	[43]
K-ISE-dPAD	10^−4^~10^−1^	-	10^−5^	[44]
SPE/OTS-Ti_3_C_2_/AuNPs/K-ISM	10^−5^~10^−1^	15	10^−5.2^	This work

**Table 2:** Comparison of different methods for determination of serum K^+^ concentration in dairy cows.

No.	Added (mM)	Measured (mM)	Recovery (%)	RSD (%)
1	-	4.8 ± 0.3	-	2.9
2	5	9.6 ± 0.4	98.0	5.8
3	10	14.4 ± 0.6	97.3	6.5
4	15	19.1 ± 0.3	96.5	3.3
5	20	24.6 ± 0.6	99.2	6.2

## Data Availability

Data are contained within the article and Supplementary Materials.

## Supplementary Materials

The following supporting information can be downloaded at: https://www.mdpi.com/article/10.3390/ijms252313204/s1. Refs. [45,46] are cited in the Supplementary Materials.
