# Supplementary material for: Application of a Screen-Printed Ion-Selective Electrode Based on Hydrophobic Ti3C2/AuNPs for K+ Determination Across Variable Temperatures
Source: Int J Mol Sci. 2024 Dec 8;25(23):13204. doi: 10.3390/ijms252313204 (PMC11641893; doi:10.3390/ijms252313204)
Supplement: Supplementary file 1 [file ijms-25-13204-s001.zip › ijms-3315386-supplementary.pdf]

Application of a Screen-Printed Ion-Selective Electrode Based on Hydrophobic Ti_3C_2/AuNPs for K^+ Determination Across Variable Temperatures

Zhixue Yu [1,2,†], Hui Wang [1,†], Yue He [1,3], Dongfei Chen [4], Ruipeng Chen [1], Xiangfang Tang [1], Mengting Zhou [1], Junhu Yao [2,*] and Benhai Xiong [1,*]

1 State Key Laboratory of Animal Nutrition, Institute of Animal Sciences, Chinese Academy of Agricultural Sciences, Beijing 100193, China; 15831207256@163.com (Z.Y.); wanghui10@caas.cn (H.W.); heyueh@163.com (Y.H.); chenruipeng@caas.cn (R.C.); tangxiangfang@caas.cn (X.T.); zhoumengting@caas.cn (M.Z.)
2 College of Animal Science and Technology, Northwest A&F University, Yangling 712100, China
3 College of Animal Science and Technology, China Agricultural University, Beijing 100193, China
4 Graduate School of Biomedical Engineering, The University of New South Wales, Sydney, NSW 2052, Australia; dongfei.chen@unsw.edu.au
* Correspondence: yaojunhu2008@nwafu.edu.cn (J.Y.); xiongbenhai@caas.cn (B.X.)
† These authors contributed equally to this work.

Interference test

The photosensitivity of the electrodes was studied. In the photosensitive test, the electrode potential value was continuously measured, and the change in potential over time is shown in Figure S1A. Before the experiment began, the electrodes were placed in darkness. After that, the electrodes were exposed to 300 s of room light, UV light, and infrared light each. The potential was recorded in 0.01 M KCl solution. No significant potential drift was observed during the measurement.

Figure S1B shows the potential response of K-ISE to the entry of O_2 and CO_2 into KCl solutions of different concentrations. It is clear that the potential did not change significantly. This could be because there was no water layer between the hydrophobic Ti_3C_2/AuNPs and K-ISM, and CO_2 would not react with water to generate carbonic acid, which would have changed the electrode's pH and potentially compromised its stability [45]. In addition, the lack of redox-active substances prevents any reaction with oxygen. In summary, light, O_2, and CO_2 had no significant effect on the potential response. It can be seen that the fabricated K-ISE with Ti_3C_2/AuNPs as the transduction layer showed good potential stability.

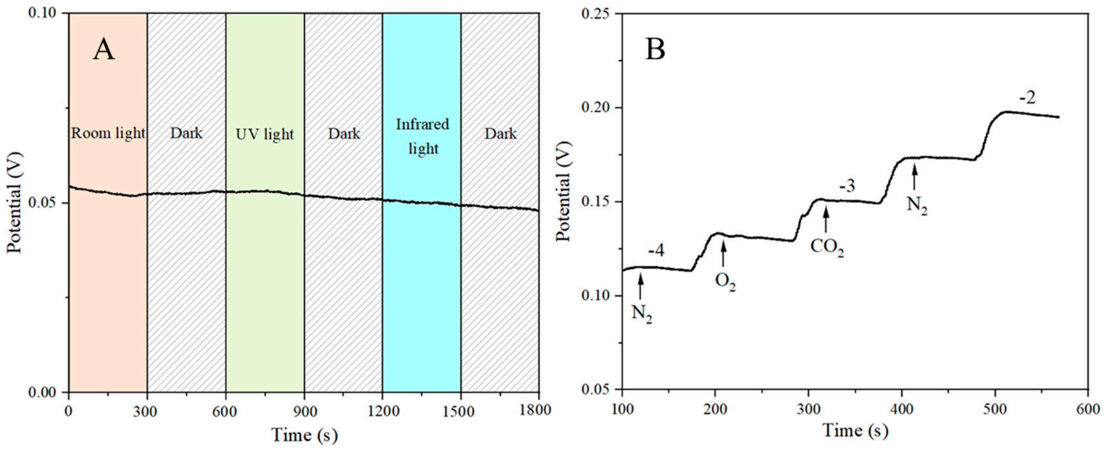

Figure S1. (A) The effect of different lights on the electrode potential stability of SPE/OTS-Ti_3C_2/AuNPs/K-ISM in 10^{-2} M KCl solution; (B) effect of O_2 and CO_2 on electrode potential stability of

SPE/OTS-Ti_3C_2/AuNPs/K-ISM in a 10^{-2} M KCl solution.

Optimization of AuNPs concentration

The amount of AuNPs deposited on the electrode surface also has an effect on the electrochemical performance. AuNPs were deposited on the electrode surface by cyclic voltammetry, and the deposition amount accounted for about 10% of the electrode surface. The deposition process is shown in Figure S3. Figure S2 shows the current variation in the SPE/OTS-Ti_3C_2/AuNPs/K-ISM under different scanning cycles. The sensor current increased from 2 r to 8 r with the increase in the scanning cycle, and decreased slightly when the scanning cycle was more than 10 r. This is because the number and volume of AuNPs increased with the increase in the scanning period, which is not conducive to electrochemical catalysis [46]. It can be seen that the sensitivity of 8 r AuNPs deposited on the electrode surface was the highest, so the 8 r AuNPs deposited on the electrode surface were selected for the experiment.

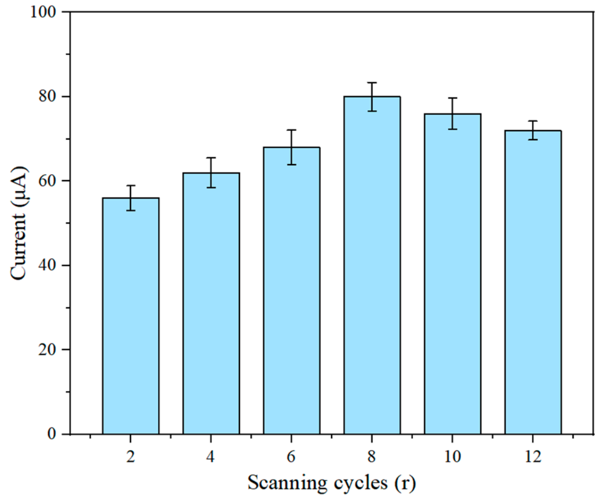

Figure S2. Current comparison of SPE/OTS-Ti_3C_2/AuNPs/K-ISM by electrodeposition of different scanning cycles of AuNPs(2 r, 4 r, 6 r, 8 r, 10 r and 12 r).

The process of AuNPs deposition

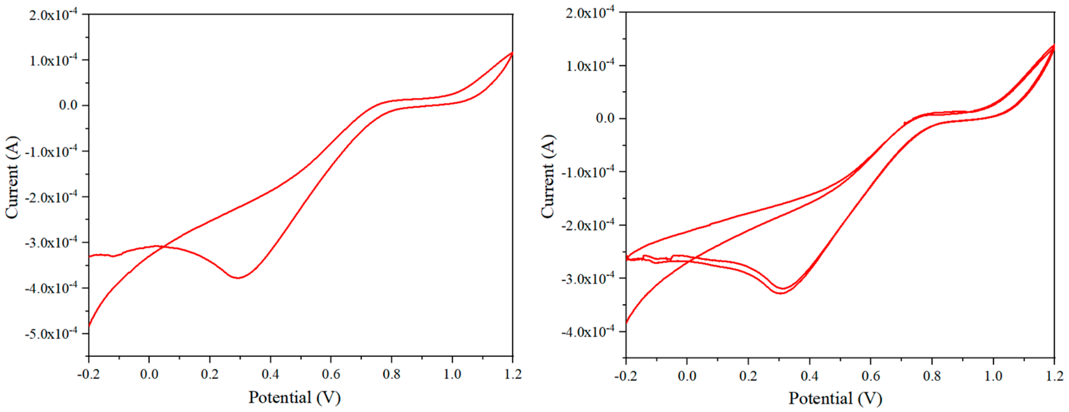

(a) (b)

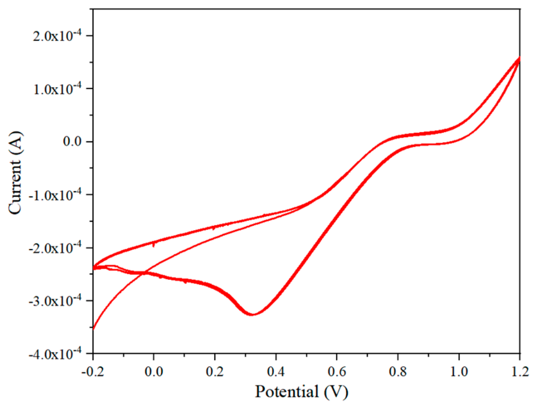

(c)

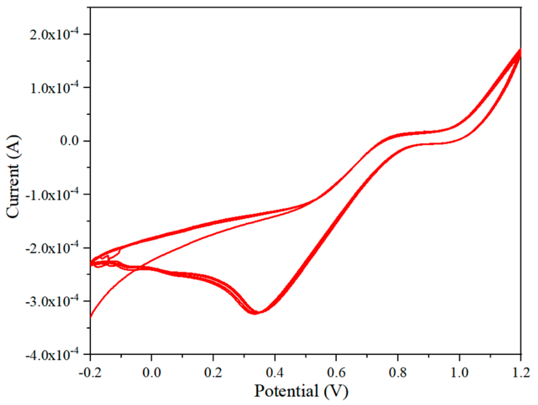

(d)

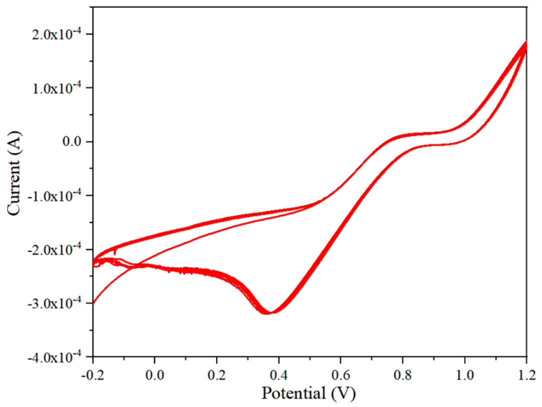

(e)

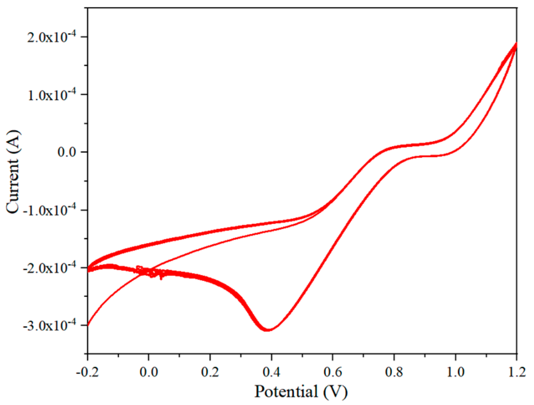

(f)

Figure S3. The images of AuNPs deposited 2 r (a), 4 r (b), 6 r (c), 8 r (d), 10 r (e), and 12 r (f).
